# Unveiling *Fusobacterium nucleatum*’s role in colorectal cancer: the inflammatory microenvironment connection

**DOI:** 10.3389/fimmu.2026.1747198

**Published:** 2026-08-12

**Authors:** Yan Chen, Xin Lu, Ke Liu, Xuejun Wen, XiaoYu Du, Yin Zhang, YingYing Kang, Ping Wang, Ying-Jian Zhang

**Affiliations:** 1 Henan Medical Key Laboratory of Gastrointestinal Microecology and Hepatology, Department of Gastroenterology, The First Affiliated Hospital, College of Clinical Medicine, Henan University of Science and Technology, Luoyang, Henan, China; 2 International Institute for Biomedical Biomaterials (I2BM), Zhengzhou, China; 3 Department of Chemical and Life Science Engineering, School of Engineering, Virginia Commonwealth University, Richmond, VA, United States; 4 Department of Public Health, School of Medicine, Henan University of Science and Technology, Luoyang, Henan, China

**Keywords:** colorectal cancer, colorectal inflammatory microenvironment, *Fusobacterium nucleatum*, myeloid-derived immune cells, NLRP3

## Abstract

**Background:**

*Fusobacterium nucleatum* (Fn), a gram-negative oral bacterium, has been implicated in promoting colorectal cancer (CRC) progression by modulating the inflammatory microenvironment. Recent studies have shown that gut microbiota imbalance plays a crucial role in CRC development, and Fn, specifically, has been found to exist in the intestine and exert pro-tumorigenic effects. However, the precise mechanisms underlying Fn-induced CRC progression remain elusive.

**Methods:**

A systematic experimental strategy was adopted to explore the function of Fusobacterium nucleatum (Fn) in colorectal cancer (CRC) progression. Tissue specimens were collected from healthy volunteers, patients with ulcerative colitis (UC), colorectal adenoma (CRA) and CRC at the First Affiliated Hospital of Henan University of Science and Technology via colonoscopy and surgical resection. Two CRC mouse models, subcutaneous MC38 model and AOM-DSS orthotopic model, were constructed, followed by intratumoral injection or oral gavage of Fn, Streptococcus mutans (S.M.) or normal saline. qPCR was used to quantify Fn abundance in colorectal tissues. IHC and FISH detected inflammatory cytokines and immune cells in tumor tissues. 16S rRNA amplicon sequencing analyzed Fn-mediated alterations in mouse gut microbiota. Flow cytometry and ELISA evaluated immune cell subsets and inflammatory cytokine levels. Western blot examined NLRP3 inflammasome and other proteins expression.

**Results:**

In this study, we employed two mouse models of CRC (MC38 subcutaneous and AOM-DSS orthotopic models) to investigate the role of Fn in CRC progression. Our findings demonstrated that Fn administration significantly increased tumor growth compared to controls. Quantitative PCR confirmed elevated Fn abundance in CRC tissues, and microbiome analysis revealed distinct bacterial clades in Fn-treated mice. Immunofluorescence analysis showed enrichment of myeloid cells (CD45/CD11b, CD11C, F4/80,MHCI,MHCII) in tumors, suggesting that Fn reshapes the immune landscape towards a proinflammatory state. *In vitro* and *in vivo* experiments further revealed that Fn selectively expands myeloid-derived immune cells. Critically, Fn failed to induce tumor growth in NLRP3^-/-^ mice, confirming that NLRP3 inflammasome activation is a key mechanism in Fn-induced CRC progression.

**Conclusion:**

Our results establish Fn as a driver of CRC progression through bone marrow-derived immune cell recruitment and NLRP3-dependent inflammatory microenvironment induction. These findings provide new insights into the mechanisms by which Fn contributes to CRC development and suggest potential therapeutic targets for CRC prevention and treatment.

## Graphical Abstract

**Figure:**
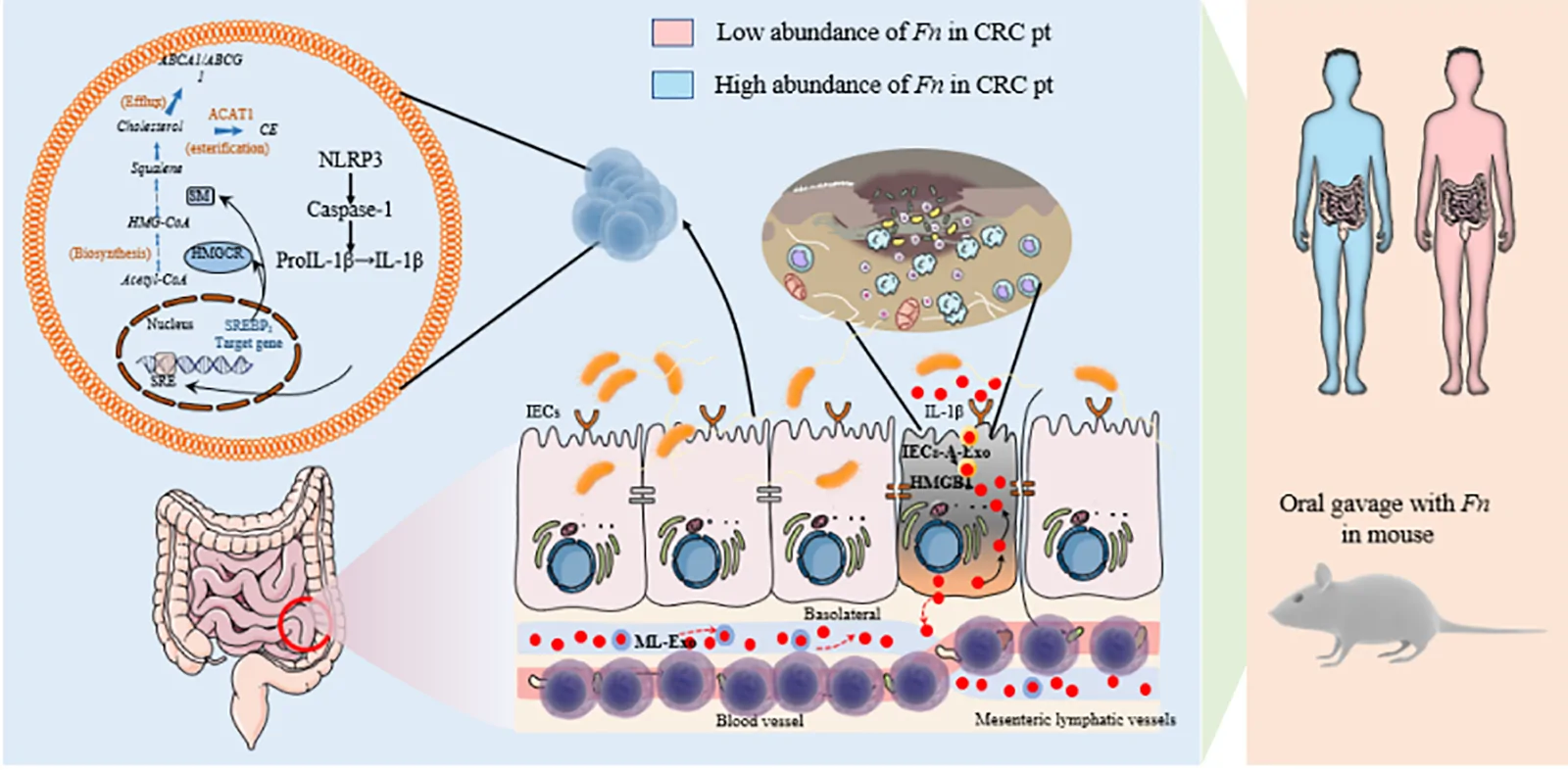

## Introduction

Intestinal flora homeostasis is essential for maintaining health and preventing disease. Dysbiosis of the gut microbiota has been linked to various human diseases, including metabolic syndrome, obesity-related disorders, liver disease, inflammatory bowel disease (IBD), and colorectal cancer (CRC) (1). Increasing evidence shows that intestinal flora imbalance plays a significant role in the occurrence, progression, and metastasis of CRC. Some studies also found microbiome alterations in colorectal adenomas (2). Therefore, changes in the microbiome may serve as biomarkers for early CRC detection. In 1969, Aries et al. first proposed a correlation between intestinal microbes and CRC. In recent years, some bacteria have been identified as pro-cancerous, among which *Fusobacterium nucleatum* (Fn) has attracted significant attention. Fn is a Gram-negative oral symbiotic bacterium commonly found in the human oral cavity, upper respiratory tract, and genital area. It is also one of the most frequently isolated microorganisms from clinical samples (3). Multiple studies confirmed the presence of Fn in the intestine, where it causes DNA damage to colorectal mucosal cells, induces chronic intestinal inflammation, suppresses host immune responses, and increases CRC incidence and poor prognosis (4). Studies have shown that exposure to an Fn-rich environment in a spontaneously tumorigenic mouse model promotes adenoma formation in the small intestine and colon, and accelerates progression to adenocarcinoma (5). However, the precise mechanisms by which Fn promotes colorectal carcinogenesis remain incompletely understood.

Inflammation is an adaptive host immune response. Changes in intestinal flora significantly influence inflammation, thereby affecting CRC development (6). Under normal conditions, intestinal epithelial cells form a single-layer mucosal barrier maintained by tight junctions, isolating intestinal flora from immune cells. During chronic inflammation, the intestinal mucosal barrier is disrupted, increasing permeability and allowing symbiotic and pathogenic microorganisms to invade intestinal tissues. Some bacteria aggregate and anchor to intestinal epithelial cells, disrupting cytoskeletal structures and breaking down epithelial connections (7). Nucleotide-binding oligomerization domain-like receptor proteins (NLRPs), also known as NALPs, are a family of inflammatory proteins extensively studied in recent years. They play critical roles in programmed cell death, inflammation, and tumor progression (8). The NLRPs family regulates multiple signaling pathways, including NF-κB and MAPK, thereby influencing tumor biology. These proteins recognize pathogen-associated molecular patterns (PAMPs) and damage-associated molecular patterns (DAMPs), playing key roles in host defense against bacterial, fungal, and viral infections, as well as tissue repair. Notably, NLRPs family members have been identified as important regulators of gastrointestinal inflammation. However, the specific mechanisms by which this family influences gastrointestinal inflammation and tumor progression require further research and evaluation (9, 10). Currently, NLRPs-related molecules have been found in human cells and more than 34 types in animal cells such as mice (11). These molecules are essential for assembling inflammasomes (8). NLRPs proteins can function as tumor suppressors during tumor development (12). However, cancer-promoting effects of NLRPs have also been reported in various cancers (13). Although previous studies reported Fn enrichment, NLRP3 inflammasome activation, and myeloid cell infiltration in CRC separately, no study has systematically integrated these three processes into a unified signaling cascade. Most prior work relied on single mouse models or *in vitro* assays alone, lacking simultaneous validation in subcutaneous and orthotopic *in vivo* models. Furthermore, direct evidence demonstrating that NLRP3 knockout completely abolishes Fn-driven CRC growth is still missing. This study fills these gaps by establishing an integrated Fn-NLRP3-myeloid cell-IL-1β signaling axis, validated across multiple models.

Therefore, this study aimed to investigate the mechanisms through which Fn promotes colorectal carcinogenesis, particularly its role in recruiting immune cells and modulating the inflammatory tumor microenvironment, including the involvement of the NLRP3 inflammasome.

## Methods

Patients: Healthy subjects undergoing physical examinations and patients with ulcerative colitis (UC), colorectal adenoma (CRA), or CRC were enrolled from the Departments of Gastroenterology and Gastrointestinal Surgery at the First Affiliated Hospital of Henan University of Science and Technology from September 2019 to December 2021. The study included 22 healthy controls, 18 UC patients, and 25 CRA patients. Tissue specimens from these participants were collected via colonoscopy. In addition, 23 CRC patients underwent surgical tissue collection, including cancer tissue from the tumor center and normal colon mucosa from 1 cm away from the tumor margin. Informed consent was obtained from all patients and their families. The study was approved by the Medical Ethics Committee of the First Affiliated Hospital of Henan University of Science and Technology.

Human Specimen Collection: Patients were selected from surgical cases at the Department of Gastrointestinal Surgery, First Affiliated Hospital of Henan University of Science and Technology. Inclusion criteria were: no prior antibiotic or chemoradiotherapy treatment before surgery, pathological confirmation by at least two pathologists, and no history of mental illness. Exclusion criteria included patients with other malignant tumors or severe organic diseases. Fresh CRC tissue and matched adjacent non-tumor tissue were collected from each patient.

Patient Clinicopathologic Characteristics: All 23 enrolled CRC patients were treatment-naïve, with no prior chemotherapy, radiotherapy, or antibiotic use. The cohort included 13 males and 10 females, with a mean age of 64.2 ± 8.1 years (range: 48–79 years). Tumors were staged postoperatively according to the 8th edition of the American Joint Committee on Cancer (AJCC) TNM staging system: stage I (n=4), stage II (n=10), stage III (n=8), and stage IV (n=1). Tumor locations included the proximal colon (n=8), distal colon (n=10), and rectum (n=5).

Sample Processing and Handling: Immediately after surgical resection, fresh tissue specimens were transported on ice to the pathology department within 30 minutes. Under pathologist guidance, tumor tissues were precisely dissected from the tumor center, and adjacent normal mucosa was obtained from sites at least 1 cm away from the tumor margin. All tissues were immediately snap-frozen in liquid nitrogen and stored at -80 °C for subsequent molecular analyses.

Animal Care: All animal experiments were approved by the Institutional Animal Care and Use Committee (IACUC) of Jiangsu Collection Medicine Kang Biotechnology Co., Ltd.

AOM-DSS-Induced Orthotopic CRC Model: Seventy-two male C57BL/6J mice (5–7 weeks old) received intraperitoneal injections of AOM (12.5 mg/kg) on Day 0. After one week, mice were randomized into three groups (n=24 each) and underwent three cycles of 2.5% DSS in drinking water (7 days DSS followed by 10 days normal water). From Day 8, mice received oral gavage twice weekly with normal saline (control), *S. mutans* (1×10 CFU), or Fn (1×10 CFU) for 8 weeks. The experiment concluded at 18–20 weeks post-AOM injection, with humane endpoints for severe distress.

MC38 Subcutaneous Xenograft Model: MC38 cells (5×10^5^) were injected subcutaneously into male mice. Once tumors reached approximately 3–4 mm in diameter, mice were randomized into three groups (n=8 each) and received intratumoral injections twice weekly with normal saline (control), *S. mutans* (1×10 CFU), or Fn (1×10 CFU) for 8 weeks. Tumor volume and body weight were monitored twice weekly.

Generation of NLRP3 Knockout Mice: NLRP3 knockout male mice were generated using CRISPR-Cas9 technology. Briefly, gRNAs targeting intronic regions of the NLRP3 gene were designed, and Cas9 with gRNAs was microinjected into fertilized eggs. Positive F0 mice were bred with C57BL/6JGpt mice to produce heterozygous offspring. The heterozygous offspring were subsequently intercrossed to generate NLRP3^+/+^, NLRP3^+/−^, and NLRP3^−/−^ mice. Genotypes were confirmed by PCR, and only male NLRP3^+/+^ and NLRP3^−/−^ mice were included in the functional experiments. NLRP3^+/−^ mice were used only for colony breeding and were not included in the reported analyses.

qPCR: Total RNA was extracted from tissues using Trizol reagent, and cDNA was synthesized via reverse transcription. Fn abundance was quantified using specific primers (Forward: 5’-ACTTACAGGTGGATGAAGGATT-3’; Reverse: 5’-CGCTCTACGTAGGAGTCTGGAC-3’) with β-actin as the endogenous control. qPCR reactions were performed in triplicate, and relative expression levels were calculated using the 2^-ΔΔCt^ method.

IHC Staining: Immunohistochemistry (IHC) staining was employed to detect NLRP3, TNF-α, IL-6, and IL-1β. Tissue sections were deparaffinized and rehydrated, followed by antigen retrieval in citrate buffer. Sections were incubated with antibodies: anti-NLRP3 (cat# GB114320, Servicebio), anti-TNF-α (cat# bs-0078R, Bioss), anti-IL-1β (cat# bs-0812R, Bioss), and anti-IL-6 (cat# bs-0752R, Bioss).

FISH: Fluorescence *in situ* hybridization (FISH) was employed to detect CD45/CD11b, CD11C, CD68, MHCI, and MHCII expression. Sections were stained with antibodies against CD45 (cat# GB115428, Servicebio), CD11b (cat# GB15058, Servicebio), CD68 (cat# GB113150, Servicebio), CD11C (cat# GB11059, Servicebio), MHCI (cat# GB150092, Servicebio), and MHCII (cat# GB150093, Servicebio). Images were analyzed using a fluorescence microscope.

16S rRNA Amplicon Sequencing and Analysis: Fecal samples were collected at three time points, with 10-day intervals. Fresh feces were aseptically collected in a laminar flow hood immediately after defecation, transferred to sterile cryotubes, placed on dry ice, and stored at -80 °C within 2 hours until DNA extraction. 16S rRNA sequencing was performed at the Microbial Genome Research Center (IMCAS, Beijing, China).

Flow Cytometry: Single-cell suspensions were incubated with Fc blocking antibody (BioLegend), stained using Zombie NIR Fixable Viability Kit (BioLegend), and labeled with surface marker antibodies. For intracellular cytokine staining, cells were stimulated with PMA (50 ng/mL, Sigma-Aldrich) and ionomycin (5 μg/mL, Sigma-Aldrich) in the presence of brefeldin A (BioLegend), followed by fixation and permeabilization. Antibodies used included anti-CD45 (BioLegend, cat#103140), anti-Ly-6G (BD, cat#562737), anti-Ly-6C (BD, cat#553104), anti-CD11b (BD, cat#550993), anti-CD11c (BioLegend, cat#117339), anti-MHC II (BioLegend, cat#107628), and anti-F4/80 (eBioscience, cat#17-4801-82). Absolute cell counts per gram of tumor tissue were calculated by multiplying each immune subset percentage by the total live cell count.

ELISA: Human THP-1 monocytic cells (CL-0233, Wuhan Punocel) were cultured in logarithmic phase. Fn standard strain ATCC 25586 (Henan Provincial Tumor Epigenetics Laboratory) was cultured anaerobically (5% CO_2_, 10% H_2_, 85% N_2_) in BHI medium for 5–7 days. THP-1 cells were divided into control (PBS) and Fn infection groups. Cells were infected with Fn at MOI 25 for 3–24 h. Cell viability remained >90% during co-culture (confirmed by trypan blue exclusion assay). Supernatants were collected at 3, 6, 12, and 24 h for cytokine analysis.

TCGA Datasets: Transcriptional expression data of NLRP3, IL-6, IL-1β, and TNF-α and corresponding clinical information were downloaded from the official TCGA website (Tomczak et al., 2015). Cancer types with at least five normal samples were included. RNA-Seq gene expression data in FPKM format were transformed into TPM and log2-transformed for further analysis. As all data were sourced from TCGA, ethical approval was not required.

Western Blot (WB): Total protein was extracted by adding pre-chilled RIPA lysis buffer containing protease inhibitors, followed by homogenization and incubation on ice. Lysates were centrifuged, and supernatants stored at -80 °C. Protein quantification was performed. Samples (25 μg total protein per well) were loaded onto SDS-PAGE gels (stacking and resolving gels prepared beforehand). Proteins were transferred via wet transfer and blocked at room temperature using 5% non-fat milk. Membranes were incubated overnight at 4 °C with primary antibodies, washed in TBST, and incubated with secondary antibodies at room temperature. After additional washes in TBST, membranes were developed using enhanced chemiluminescence (ECL). The antibodies used were anti-pro Caspase-1 (cat# ab179515, Abcam), anti-Caspase-1 (cat# 14-9832-80, Thermo), anti-IL-1β (cat# NB600-633, NOVUS), anti-NLRP3 (cat# SAB5700723, Merck), anti-α-Tubulin (cat# BS1699, Bioworld), and anti-GAPDH (cat# MA5-35235, Thermo). Protein expression was normalized to loading controls (α-Tubulin or GAPDH), as indicated in figure legends.

Statistical Analysis: Statistical analyses were performed using PASW Statistics 18 (SPSS Inc.) and GraphPad Prism 6 (GraphPad Software, Inc.). Data are presented as the mean ± SEM unless otherwise indicated. Data distribution was assessed for normality before statistical testing. For comparisons between two independent groups, an unpaired two-tailed Student’s *t*-test was used for normally distributed data, whereas the Mann–Whitney *U* test was used for non-normally distributed data. For paired patient samples, a paired Student’s *t*-test or Wilcoxon matched-pairs signed-rank test was used, as appropriate. Comparisons among three or more groups were performed using one-way ANOVA followed by Tukey’s multiple-comparisons test. For non-normally distributed data involving three or more groups, the Kruskal–Wallis test followed by Dunn’s multiple-comparisons test was used. Two-way ANOVA followed by an appropriate multiple-comparisons test was used for experiments involving two independent factors. Longitudinal measurements obtained from the same animals, including body weight, tumor growth, and DAI scores, were analyzed using two-way repeated-measures ANOVA. Categorical data were analyzed using the χ² test or Fisher’s exact test. All tests were two-sided, and *P* < 0.05 was considered statistically significant. Statistical significance was defined as follows: *P < 0.05, **P < 0.01, ***P < 0.001, and ****P < 0.0001.

## Results

### Fn is enriched in CRC patient samples

**Figure 1:**
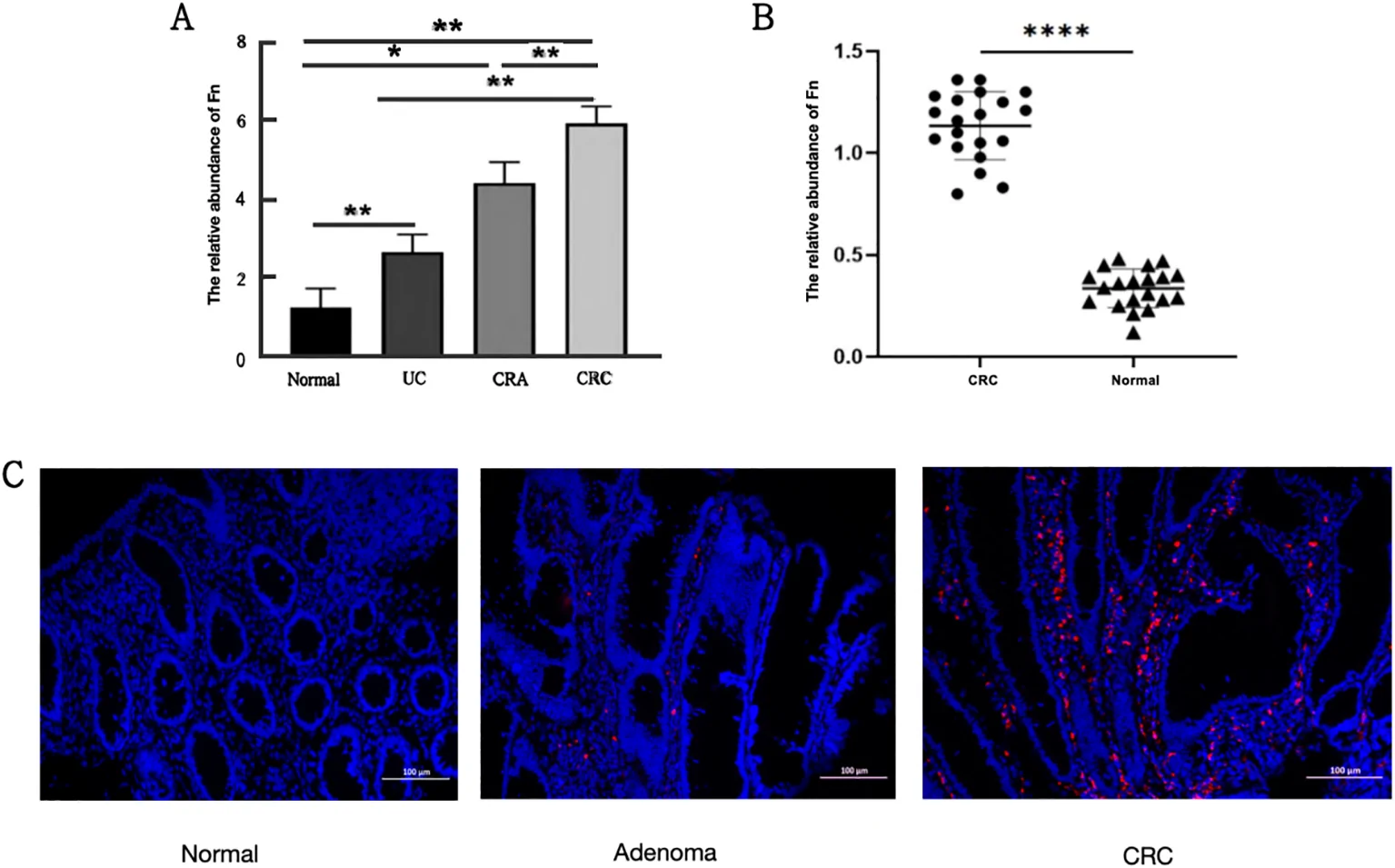
Fn was detected at significantly higher abundance in tissues from CRC patients compared with adenoma patients and healthy controls, and was enriched in carcinoma tissue compared to adjacent normal tissue. **(A)** Fn abundance in different colorectal tissues. **(B)** Fn abundance in CRC versus adjacent normal tissues. **(C)** Representative FISH images of Fn in normal controls, adenoma tissues, and CRC samples using a Cy3-conjugated Fn 16S rDNA-directed probe (red; Fn colonization, magnification 200×) (*P < 0.05, **P < 0.01, ****P < 0.0001).

qPCR was employed to analyze and compare Fn abundance across different colorectal tissues. Fn abundance was significantly higher in the CRC group compared with the UC, CRA, and healthy control groups (P < 0.05). Additionally, Fn abundance in the UC and CRA groups was significantly higher than in healthy controls (P < 0.05, **Figure 1A**). Next, qPCR analysis of 20 paired colorectal specimens revealed that the relative Fn abundance was 1.13 ± 0.16 in CRC tissues and 0.33 ± 0.09 in adjacent normal tissues. Fn abundance in CRC tissues was significantly increased compared with adjacent tissues (P < 0.05, **Figure 1B**). Further FISH staining confirmed significantly greater Fn abundance in CRC tissues compared to normal and adenoma tissues (**Figure 1C**). Collectively, these results demonstrate significant enrichment of Fn in CRC patients.

### Fn promotes colorectal tumor progression in a CRC xenograft mouse model

**Figure 2:**
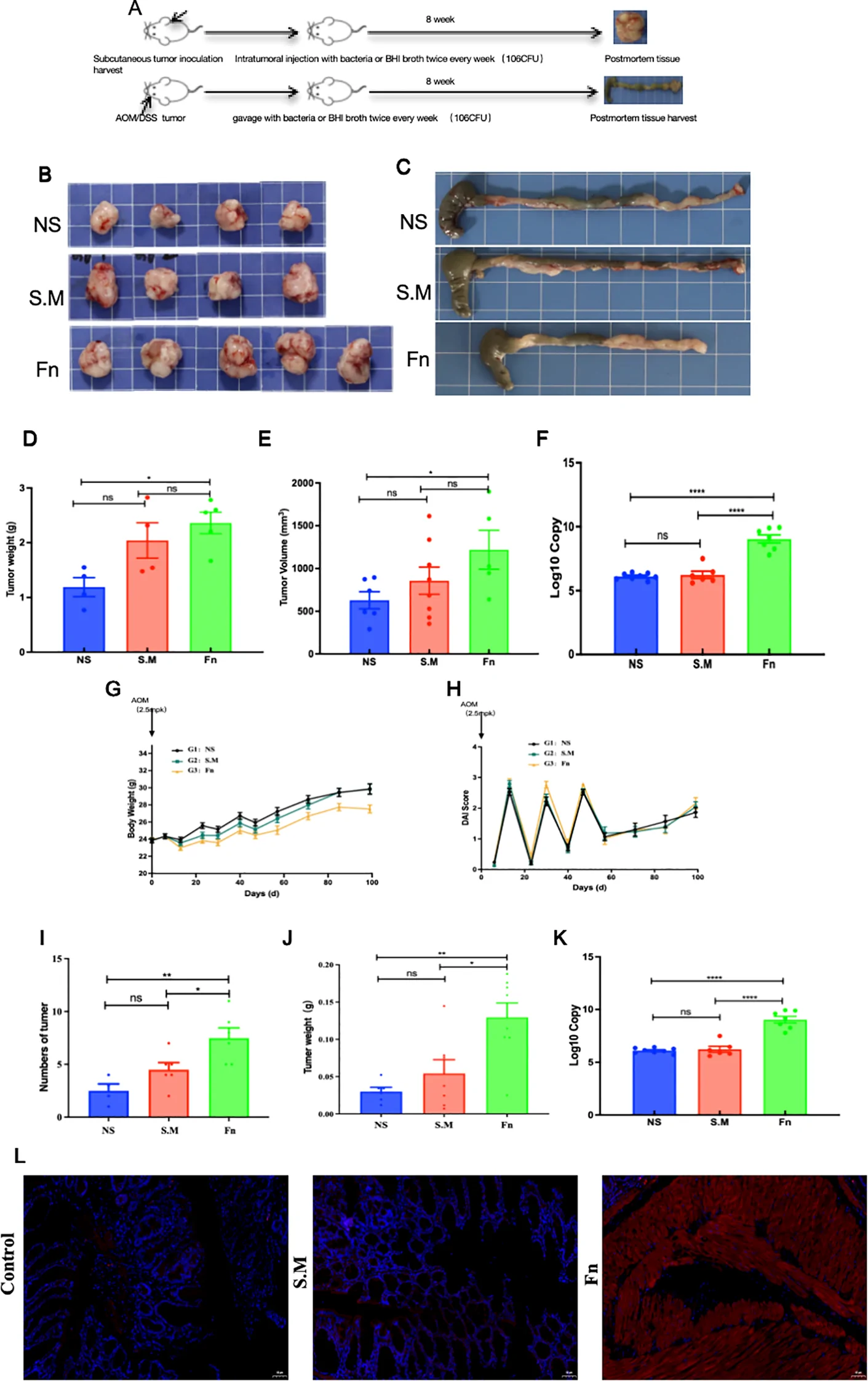
Fn promotes colorectal tumor growth in male xenograft mouse models. **(A)** Schematic diagram illustrating the experimental protocol to determine Fn’s role in promoting colorectal tumor growth in C57BL/6 mice. Two mouse models were used. At 8 weeks of age, mice received intratumoral injections of Fn in the subcutaneous MC38 tumor model or oral gavage of Fn in the AOM-DSS orthotopic rectal carcinoma model. Control mice received injections or oral gavage with *S. mutans* or BHI broth. **(B, C)** Schematic diagrams of subcutaneous and orthotopic colon cancer models. **(D, E)** MC38 tumors from mice treated intratumorally with Fn, S. mutans, or BHI broth. Tumor volume and weight are shown. Tumor volume was calculated using the formula: V = LW^2^/2. **(F)** qPCR (TaqMan probe-based) analysis of Fn abundance in tumor tissues from mice injected intratumorally with Fn, S. mutans, or BHI broth. **(G, H)** Fn induces weight loss and inflammatory changes in mice. **(I, J)** Orthotopic tumors from AOM-DSS model mice orally gavaged with Fn, S. mutans, or BHI broth. Tumor numbers and weights for each group are shown. **(K)** qPCR analysis of Fn abundance in tumor tissues from mice orally gavaged with Fn, S. mutans, or BHI broth. **(L)** FISH analyses (Cy3-conjugated Fn 16S rDNA probe) of paraffin sections from tumor tissues of mice orally gavaged with Fn, S. mutans, or BHI broth. Data represent mean ± SEM. Data involving three experimental groups were analyzed using one-way ANOVA followed by Tukey’s multiple-comparisons test, whereas longitudinal body-weight and DAI measurements were analyzed using two-way repeated-measures ANOVA followed by Tukey’s multiple-comparisons test. *P < 0.05, **P < 0.01, ***P < 0.001, and ****P < 0.0001; ns, not significant.

We explored whether Fn promotes colorectal tumor progression using two established mouse models (subcutaneous MC38 and AOM-DSS orthotopic models; experimental schema in **Figure 2A**). In the subcutaneous MC38 model (described in Methods), intratumoral administration of Fn significantly enhanced tumor growth compared with controls. Specifically, tumor weight and volume were markedly elevated in the Fn-treated group (**Figures 2B, D, E**). Furthermore, Fn enrichment in tumor tissues was confirmed by qPCR (**Figure 2F**). In the AOM-DSS orthotopic CRC model, Fn was administered orally by gavage. Fn administration resulted in weight loss and inflammation in mice (**Figures 2G, H**). As in the subcutaneous model, Fn increased tumor numbers and tumor weight in the orthotopic model (**Figures 2C, I, J**). Fn enrichment in tumor tissues of Fn-gavaged AOM-DSS mice was confirmed by qPCR and FISH (**Figures 2K, L**).

**Figure 3:**
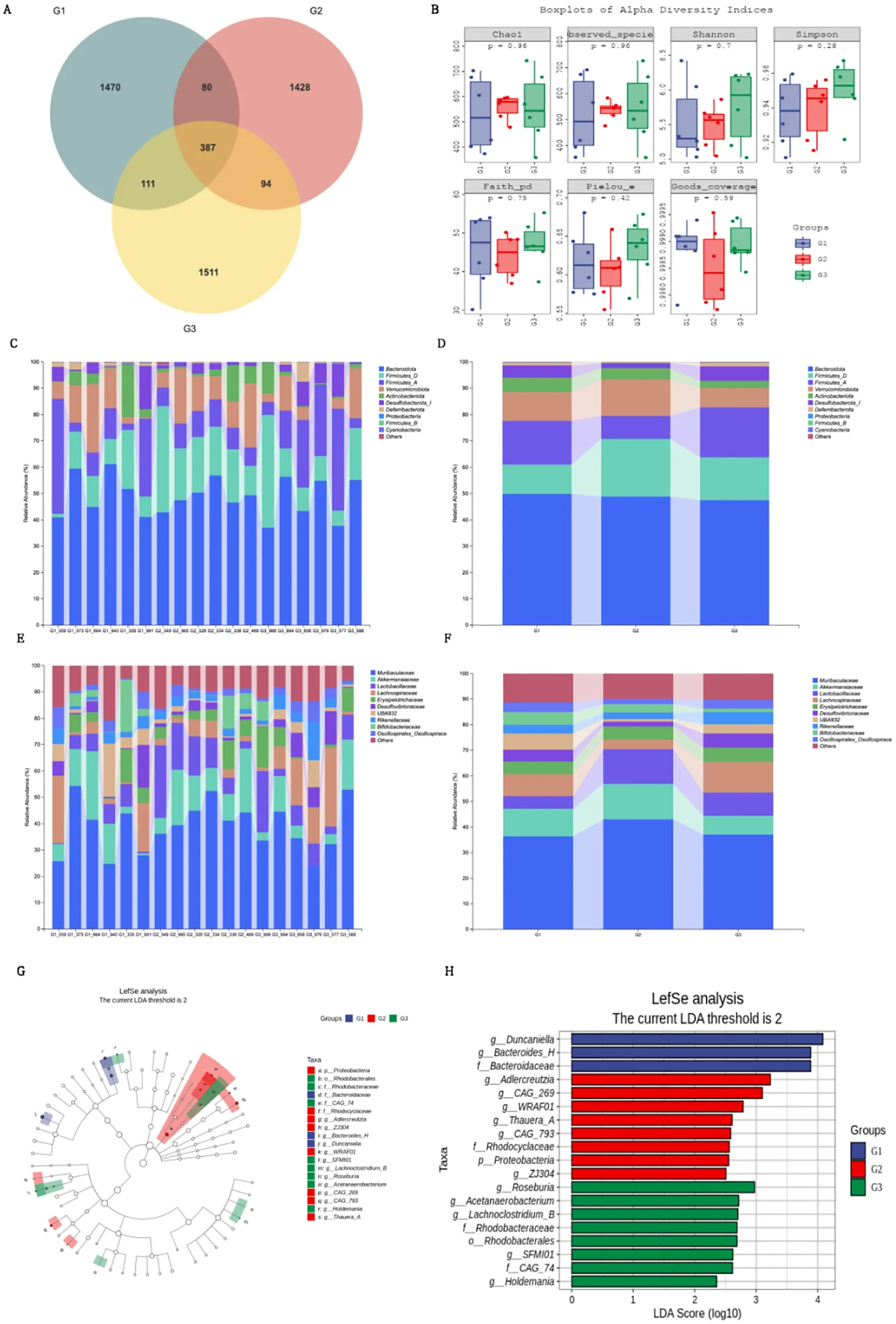
Effect of Fn on gut microbiota dysbiosis in male CRC mouse models. **(A)** Venn diagram showing average OTUs and overlaps between groups. **(B)** Boxplots of alpha diversity indices. **(C)** Microbial community structures at the phylum level in different groups. **(D)** Community bar plot of microbiota at the phylum level. **(E)** Microbial community structures at the family level in different groups. **(F)** Community bar plot of microbiota at the family level. **(G, H)** LDA effect size (LEfSe) of microbiota in different groups (G1: saline; G2: (S) mutans; G3: Fn).

**Figure 4:**
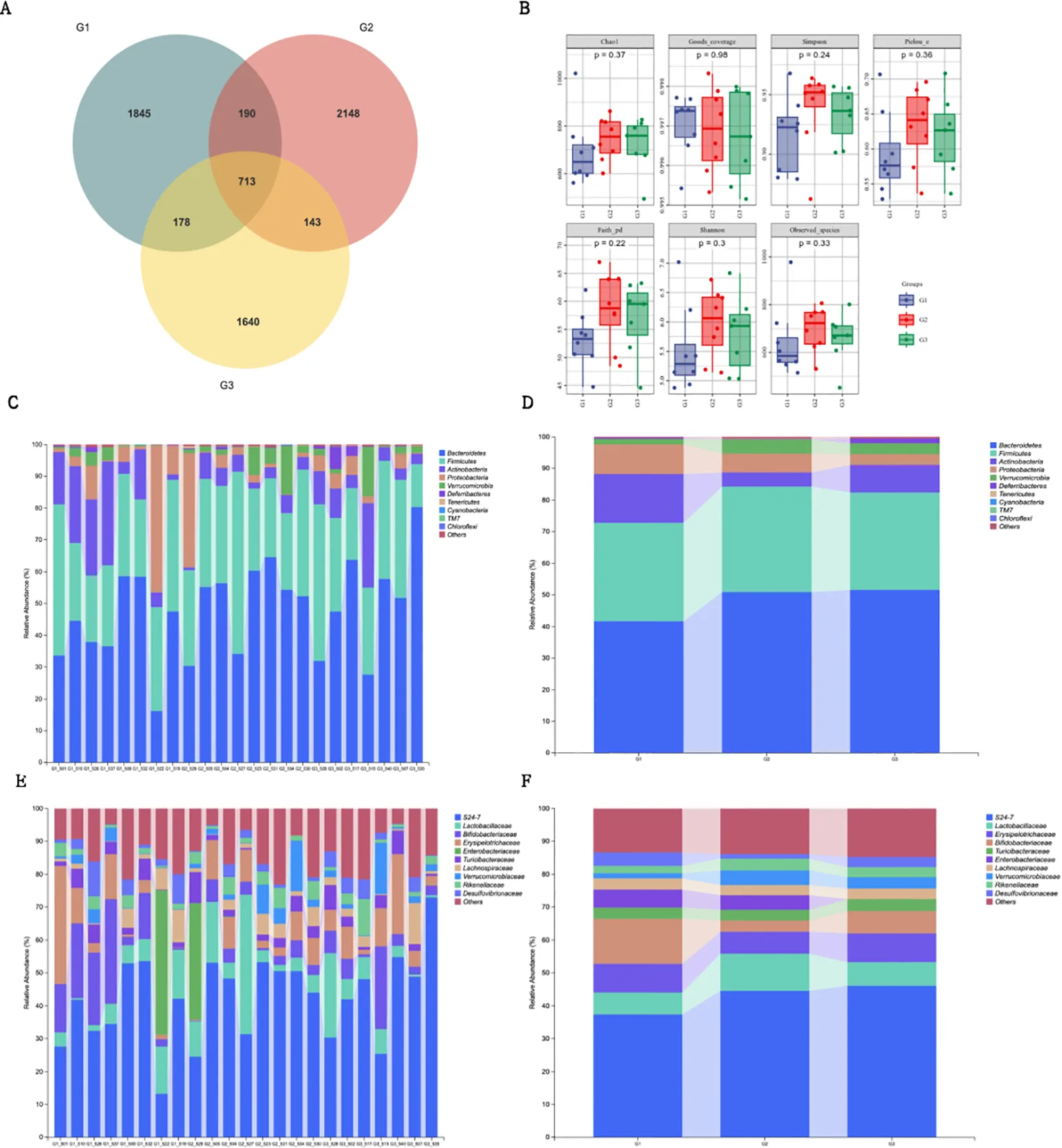
Effect of Fn on gut microbiota dysbiosis in subcutaneous MC38 male mouse models. **(A)** Venn diagram showing average OTUs and overlaps among groups. **(B)** Boxplots of alpha diversity indices. **(C)** Microbial community structures at the phylum level in different groups. **(D)** Community bar plot of microbiota at the phylum level. **(E)** Microbial community structures at the family level in different groups. **(F)** Community bar plot of microbiota at the family level (G1: saline; G2: (S) mutans; G3: Fn).

To characterize Fn’s impact on the resident gut microbiome, fecal samples from orthotopic mice were analyzed by 16S rRNA gene sequencing. A Venn diagram showed average operational taxonomic units (OTUs) and OTU overlaps among groups (G1: saline; G2: S. mutans; G3: Fn; **Figure 3A**). Alpha diversity indices indicated increased richness and evenness in the Fn group compared with the other two groups (**Figure 3B**). At the phylum level, Fn treatment increased the abundance of Firmicutes_A, Deferribacterota, and Desulfobacterota_I, while decreasing Verrucomicrobiota and Actinobacteriota (**Figures 3C, D**). At the family level, Fn treatment increased Lachnospiraceae, Rikenellaceae, and Desulfovibrionaceae, while decreasing Akkermansiaceae and Bifidobacteriaceae (**Figures 3E, F**). Linear discriminant analysis (LDA) effect size (LEfSe) demonstrated increased abundance of Roseburia, Acetanaerobacterium, Lachnoclostridium B, Rhodobacteraceae, Rhodobacterales, SFMIO1, CAG74, and Holdemania in the Fn group (**Figures 3G, H**). These data indicate that Fn alters the gut microbiome and promotes tumor growth in orthotopic CRC models. Similarly, in the subcutaneous MC38 CRC model, Fn also altered the gut microbiome and promoted tumor growth (**Figure 4**).

### Fn alters the tumor immune microenvironment by selectively expanding myeloid-derived immune cells and inducing a proinflammatory environment

**Figure 5:**
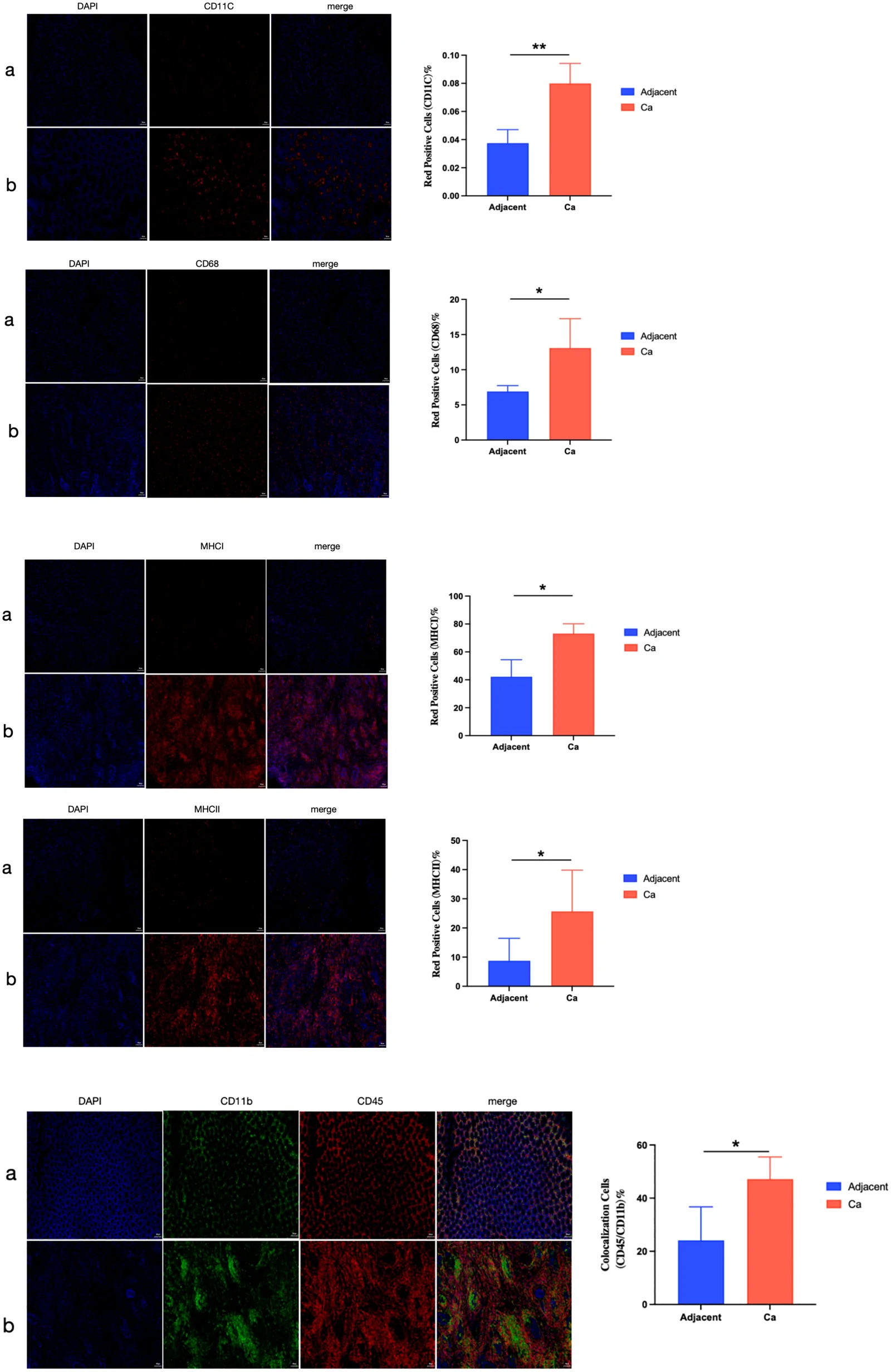
Expression of immune markers CD45/CD11b, CD11C, CD68, MHCI, and MHCII in myeloid-derived cells in CRC **(b)** and adjacent normal tissues **(a)**. *P < 0.05, **P < 0.01.

To investigate myeloid cell expression in CRC tissues, multiplex immunofluorescence assays were performed to detect immune markers CD45/CD11b (red fluorescence: CD45; green fluorescence: CD11b), CD68, CD11C, MHCI, and MHCII in different myeloid cell subsets. The results (**Figure 5**) indicated significantly higher levels of CD45/CD11b, CD11C, CD68, MHCI, and MHCII in CRC tissues compared to adjacent normal tissues (P < 0.05). These findings suggest enrichment of these myeloid cell subsets in CRC tissues.

**Figure 6:**
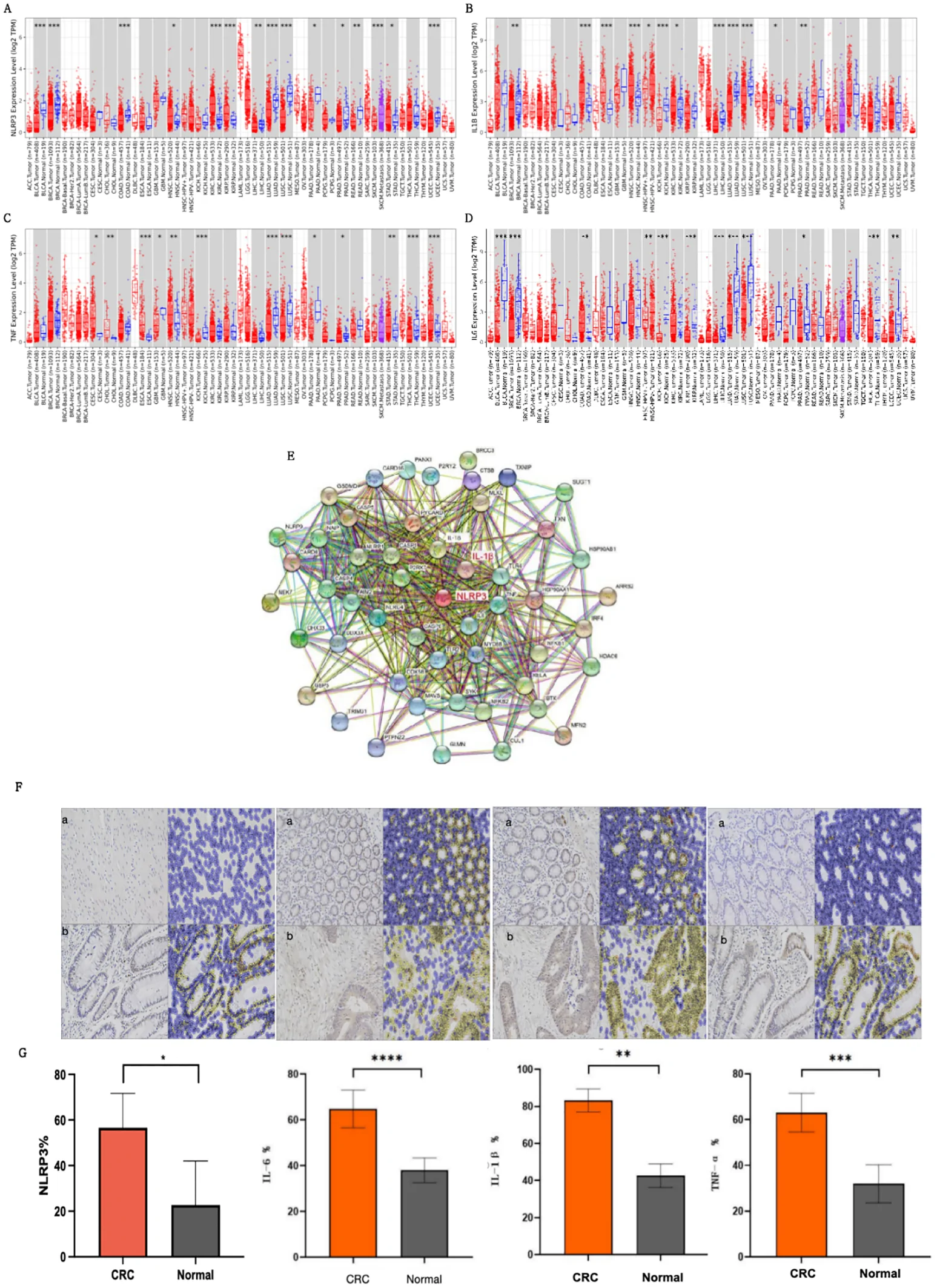
Fn promotes CRC progression by inducing a proinflammatory microenvironment. **(A–D)** Expression levels of NLRP3, IL-1β, TNF-α, and IL-6 in tumor versus adjacent normal tissues. **(E)** NLRP3-associated protein interactions. **(F)** Representative immunohistochemical staining images (400× magnification) of NLRP3, IL-6, IL-1β, and TNF-α in CRC and adjacent normal tissues. **(G)** Quantification of positively stained cells (*P < 0.05; **P < 0.01; ***P < 0.01; ****P < 0.0001).

Using the TCGA database, expression levels of NLRP3 and downstream inflammatory factors (IL-1β, TNF-α, IL-6) were analyzed in various tumor and adjacent normal tissues. The results showed significantly elevated expression of NLRP3 and IL-1β in CRC compared to normal tissues (**Figures 6A, B**), indicating their potential involvement in CRC pathogenesis. A protein-protein interaction (PPI) network constructed via the STRING database (**Figure 6E**) identified 50 proteins closely associated with NLRP3, including IL-1β. Additionally, TCGA analyses confirmed significantly higher expression of TNF-α and IL-6 in COAD and READ compared to adjacent normal tissues (P < 0.05, **Figures 6C, D**). Immunohistochemical analysis (**Figure 6F**) further validated elevated levels of NLRP3, IL-6, IL-1β, and TNF-α in collected CRC samples compared to adjacent tissues (55.37 ± 6.24% vs. 18.35 ± 4.36%, 64.78 ± 8.26% vs. 37.96 ± 5.43%, 83.20 ± 6.23% vs. 42.59 ± 6.34%, and 63.05 ± 8.46% vs. 31.94 ± 8.34%, respectively; P < 0.05, **Figure 6G**). These data indicate that Fn promotes CRC progression by inducing a proinflammatory microenvironment.

**Figure 7:**
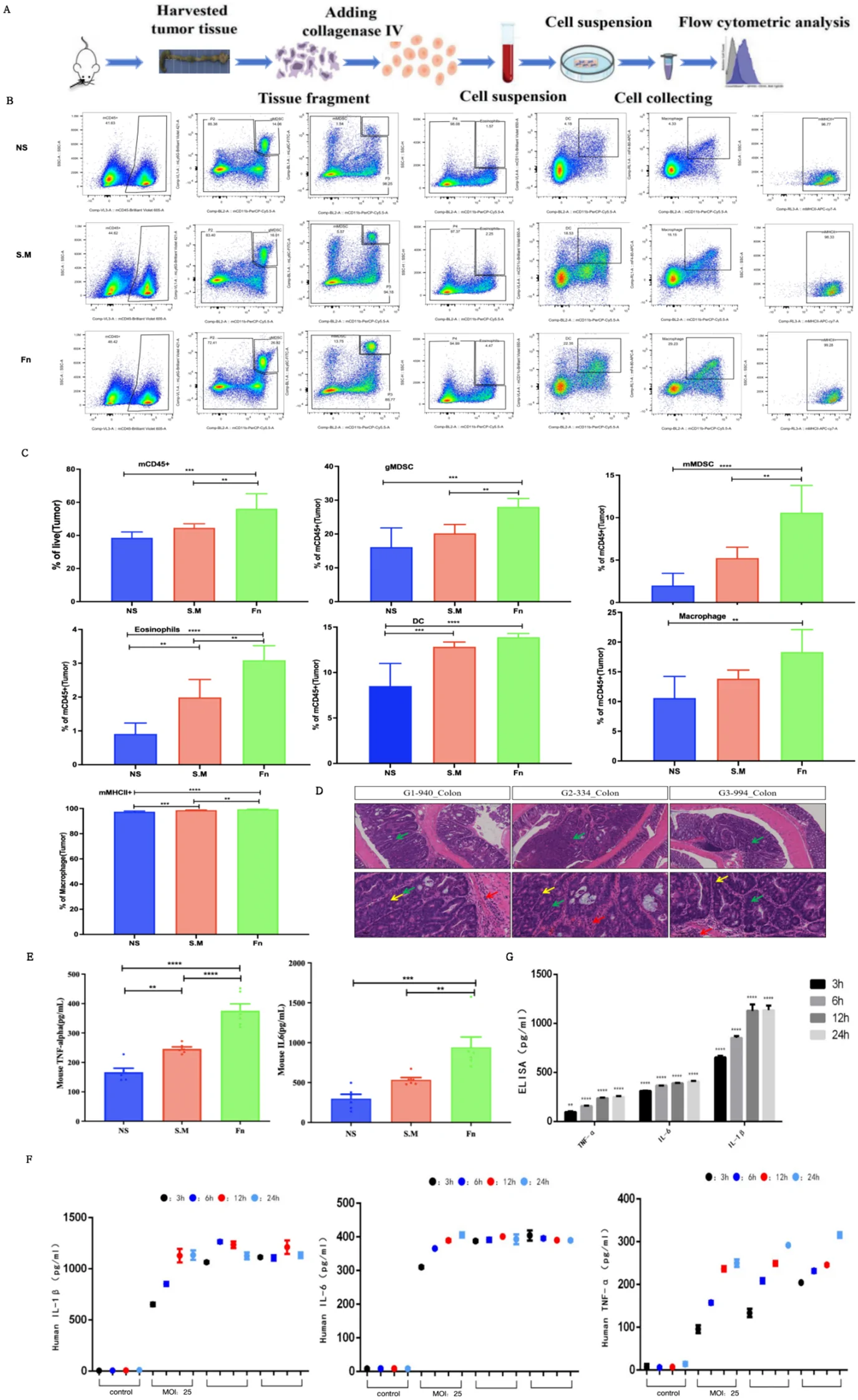
Fn alters the tumor immune environment by selectively expanding myeloid-derived immune cells and inducing a proinflammatory microenvironment. **(A)** Schematic diagram showing the process for generating single-cell suspensions from AOM-DSS orthotopic rectal carcinomas. Tumors were harvested, dissociated into single cells, and cultured (AOM-DSS model). **(B, C)** Flow cytometry analysis of CD11b+ myeloid cells, CD11b+ MHCII+ myeloid antigen-presenting cells, and differentiated myeloid cells. **(D)** Representative HE staining images. **(E)** ELISA results for IL-6 and TNF-α levels. **(F, G)** qPCR and ELISA analyses of TNF-α, IL-6, and IL-1β mRNA expression and protein secretion in THP-1 cells stimulated with Fn (MOI = 25) for 3, 6, 12, and 24 hours. Data represent mean ± SEM. Comparisons among the three animal treatment groups were performed using one-way ANOVA followed by Tukey’s multiple-comparisons test. The effects of *Fn* treatment and stimulation time in THP-1 cells were analyzed using two-way ANOVA followed by Šídák’s multiple-comparisons test. *P < 0.05, **P < 0.01, ***P < 0.001, and ****P < 0.0001; ns, not significant.

To determine if Fn contributes to tumorigenesis through effects on intratumoral immune cells and inflammation, infiltrating immune cells from AOM-DSS orthotopic rectal carcinoma mice (Fn-gavaged or BHI control) were analyzed. Tumors were dissociated into single-cell suspensions for analysis (**Figure 7A**). Fn-treated mice exhibited increased infiltration of myeloid-lineage cells, including CD11b^+^ cells, CD11b^+^ MHCII^+^ antigen-presenting cells, macrophages, dendritic cells (DC), CD45^+^, granulocytic (gMDSC), monocytic (mMDSC) myeloid-derived suppressor cells (MDSCs), and eosinophils compared to controls (P < 0.05, **Figures 7B, C**). Next, HE staining was performed on tumor tissues from the three groups. The results showed marked inflammatory cell infiltration among tumor cells in the Fn group (**Figure 7D**). ELISA was performed to detect IL-6 and TNF-α levels in tumor tissues, and the results were consistent with the pathological findings. IL-6 and TNF-α expression levels in the Fn gavage group were significantly higher than those in the other two groups (P < 0.05; **Figure 7E**). Because these findings indicated myeloid cell enrichment and a proinflammatory microenvironment, we further investigated whether Fn directly activates myeloid cells. The myeloid cell line THP-1 was infected with Fn for 3, 6, 12, and 24 h (MOI = 25) *in vitro*. Total RNA was extracted, and culture supernatants were collected after infection. TNF-α, IL-6, and IL-1β levels were measured by qPCR and corresponding ELISAs. Fn significantly increased the expression and secretion of TNF-α, IL-6, and IL-1β in THP-1 cells (**Figures 7F, G**). These results suggest that Fn may act as a potential initiator of CRC by altering the tumor immune microenvironment through recruitment of myeloid cells and induction of proinflammatory cytokines, including TNF-α, IL-6, and IL-1β. Collectively, these findings demonstrate that Fn alters the tumor immune microenvironment by selectively expanding myeloid-derived immune cells and promoting a proinflammatory microenvironment.

### Fn activates the NLRP3 inflammasome *in vitro* and fails to induce colorectal tumor growth in NLRP3^-/-^ mice

**Figure 8:**
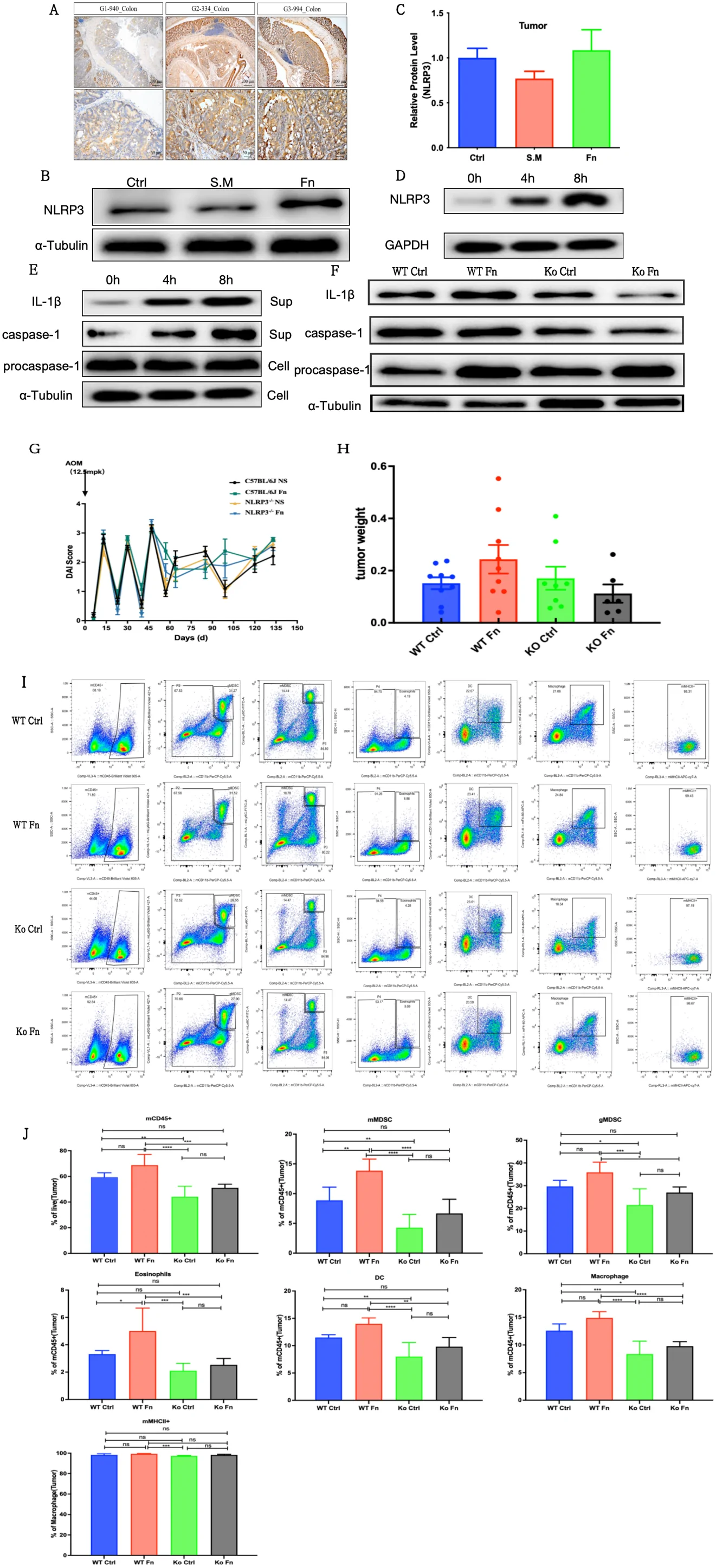
Fn activates the NLRP3 inflammasome *in vitro* but fails to induce colorectal tumor growth in NLRP3^-/-^ mice. **(A–C)** NLRP3 inflammasome expression in tumor tissues and lysates (AOM-DSS orthotopic model) was detected by IHC and WB. **(D)** WB analysis of NLRP3 protein levels in BMDMs cocultured with Fn for 4 and 8 hours. **(E)** Levels of procaspase-1 (in cells) and caspase-1 and IL-1β (in supernatants) after BMDMs were cocultured with Fn for 4 and 8 hours. **(F)** Procaspase-1 (cell lysates) and IL-1β and caspase-1 (supernatants) after coculture of Fn with NLRP3^+/+^ or NLRP3^-/-^ BMDMs for 4 and 8 hours. **(G, H)** DAI scores and body weight of each experimental group. **(I, J)** Cell numbers per gram of tumor tissue (CD11b^+^ myeloid cells, DCs, macrophages, etc.) in each group. Data represent mean ± SEM. Longitudinal DAI measurements were analyzed using two-way repeated-measures ANOVA. Comparisons involving genotype and Fn treatment were performed using two-way ANOVA followed by Tukey’s multiple-comparisons test. Comparisons among different stimulation times were performed using one-way ANOVA followed by Tukey’s multiple-comparisons test. *P < 0.05, **P < 0.01, ***P < 0.001, and ****P < 0.0001; ns, not significant.

We demonstrated that Fn activates IL-1β production and secretion in tumor tissues and cultured cells. IL-1β activation occurs via two processes: transcription of pro-IL-1β mRNA (regulated by NF-κB) and maturation of IL-1β (regulated by inflammasome complexes). Among various inflammasomes discovered, NLRP3 is the most extensively studied. To determine whether the NLRP3 inflammasome participates in Fn-induced IL-1β secretion, we measured NLRP3 inflammasome expression in tumors and lysates from AOM-DSS orthotopic rectal carcinoma models by IHC and WB. Although both Fn and *S. mutans* treatments increased tumor growth compared to saline (**Figures 2I, J**), Fn treatment exhibited a significantly stronger effect, prompting us to further investigate the inflammatory signaling associated with *Fn* under the experimental conditions used in this study. The NLRP3 inflammasome level was significantly elevated in the Fn-treated group (P < 0.05; **Figures 8A–C**). As Fn recruits myeloid cells and induces inflammation in CRC, bone marrow-derived macrophages (BMDMs) were selected for *in vitro* studies. After BMDMs were cocultured with Fn for 4 or 8 hours, NLRP3, procaspase-1, and caspase-1 protein expression in cells and IL-1β and caspase-1 secretion in supernatants were evaluated. Fn induced NLRP3, caspase-1, and IL-1β expression in a time-dependent manner, while procaspase-1 levels remained unchanged (**Figures 8D, E**). The involvement of NLRP3 in Fn-induced IL-1β secretion was further evaluated using BMDMs from NLRP3^+/+^ and NLRP3^-/-^ mice. NLRP3 knockout significantly inhibited Fn-induced caspase-1 activation and IL-1β secretion but did not alter procaspase-1 levels (**Figure 8F**).

Next, we investigated whether Fn promotes CRC through NLRP3 activation *in vivo*. Tumor weight in Fn-treated NLRP3^-/-^ mice was significantly reduced compared with NLRP3^+/+^ mice (**Figure 8H**). Disease activity index (DAI) scores are shown in **Figure 8G**. Flow cytometry of tumors from NLRP3^+/+^ mice demonstrated significant enrichment of myeloid cells in the Fn group. In contrast, no myeloid cell enrichment occurred in tumors from NLRP3^-/-^ mice (P < 0.05, P < 0.01; **Figures 8I, J**).

## Discussion

Our study investigates how *F. nucleatum* promotes colorectal carcinogenesis and provides an integrated assessment of its association with the inflammatory tumor microenvironment. Although previous studies have independently reported Fn enrichment, NLRP3 inflammasome activation, and myeloid-cell recruitment in CRC, the functional relationships among these processes have not been fully characterized within a unified experimental framework. Therefore, the contribution of the present study does not lie in identifying each individual connection as entirely novel, but in mechanistically integrating these previously recognized observations and validating their relationships across patient specimens, *in vitro* myeloid-cell assays, two complementary CRC mouse models, and NLRP3-deficient mice. Building upon the established association of Fn with CRC (2, 14), our findings support a model in which Fn-associated myeloid-cell accumulation and NLRP3 inflammasome activation contribute to IL-1β production and enhanced colorectal tumor progression, thereby extending previous observations through cross-model functional evidence. Previous research demonstrated that fecal microbiota from CRC patients can induce intestinal tumorigenesis in mice (15), validating the direct pro-tumorigenic role of the gut microbiome. Dysregulated gut microbiota in CRC reflects critical host–microbe interactions during tumor development, providing novel opportunities for cancer prevention, diagnosis, and treatment (16). Fn is consistently enriched in CRC tissues and feces and is associated with poor clinical outcomes (2), supporting its role as a significant risk factor for CRC progression.

Fn is a Gram-negative anaerobic bacterium predominantly found in the oral cavity and commonly identified in CRC tissues (3). In this study, Fn colonization and expression levels were assessed in healthy subjects, UC patients, CRA patients, and CRC patients using qPCR. Fn expression was significantly increased in CRC patients compared with matched adjacent normal tissues, consistent with previous reports. Numerous studies have demonstrated that Fn promotes CRC development through multiple mechanisms. Adhesion and invasion are crucial mechanisms by which Fn colonizes tissues, disseminates, evades host defenses, and induces host immune responses (17). Fn contributes to CRC development by interacting with multiple signaling pathways through specific biomolecules expressed on its surface, including adhesion protein A (FadA), transporter 2 (Fap2), and lipopolysaccharide (LPS) (18). These biomolecules activate immune signaling pathways, disrupt homeostasis, and establish an inflammatory microenvironment. Increased FadA levels correlate with elevated expression of oncogenes and inflammatory genes and are notably higher in colorectal adenoma and carcinoma patients. FadA binds to E-cadherin on CRC cells, mediates Fn adhesion and invasion, activates β-catenin signaling, and increases expression of Wnt pathway components and NF-κB. This cascade contributes to APC mutations and CRC progression (19). Experimental studies indicate that Fn increases DNA damage through the FadA/E-cadherin/β-catenin axis, accelerating colorectal tumor growth (20). Fap2, another adhesion protein, recognizes and binds to Gal-GalNAc molecules overexpressed on CRC cells, facilitating Fn enrichment and colonization (21). Casasanta et al. demonstrated in mice that Fap2 mediates Fn binding specifically to CRC cells, exploiting high Gal-GalNAc expression. Fap2-expressing Fn also translocates to the colorectal region through circulation, colonizes intestinal epithelium or tumors, disrupts intestinal microbiota, and promotes tumorigenesis (22). LPS from Fn activates β-catenin signaling via the TLR4/P-PAK1 pathway, enhancing NF-κB activation and CRC progression (23). Recent studies have highlighted the role of microRNAs, such as miRNA-21, in CRC pathogenesis (24). Previous researchers proposed that Fn activates the TLR4/MYD88/NF-κB signaling pathway in intestinal epithelial cells, thereby regulating miRNA-21 expression to promote colorectal tumor progression (25). These studies collectively suggest that Fn critically influences CRC pathogenesis, primarily through immune and inflammatory mechanisms. In this study, our findings demonstrate that Fn recruits tumor-infiltrating myeloid cells, activates the NLRP3 inflammasome, and establishes a proinflammatory microenvironment, collectively promoting colorectal tumor progression.

This study, based on the TCGA database, analyzed the expression of NLRP3 inflammasome and inflammatory factors IL-6, IL-1β, and TNF-α across various cancers and corresponding adjacent tissues. The results revealed significantly higher expression levels of these inflammatory factors in CRC tissues compared with adjacent normal tissues. Immunohistochemical analysis demonstrated minimal cytoplasmic IL-1β expression in colorectal glandular epithelial cells of the healthy control group. In contrast, cytoplasmic expression of IL-1β was elevated in glandular epithelial cells from UC and CRA patients, and prominently increased in CAC cells. These findings suggest these inflammatory factors as potential diagnostic biomarkers for CRC.

Mouse models of bacterial-driven intestinal tumorigenesis have been employed to confirm bacterial contributions to tumor progression (26, 27). To study both early and advanced CRC stages, subcutaneous and orthotopic rectal cancer mouse models were used. Our results demonstrated the significant role of Fn in CRC initiation and progression. Fn possesses multiple virulence factors capable of driving immune evasion, including suppression of host immunity, modulation of cytokine production, and alterations in cell signaling pathways. In this study, a significant increase in CD11b^+^ myeloid cell infiltration was observed at tumor sites in mice infected with Fn compared to uninfected controls. Myeloid cells have previously been associated with tumor progression, angiogenesis, and modulation of antitumor immunity (28–30). Additionally, we noted elevated expression of proinflammatory cytokines IL-1β, IL-6, and TNF-α, which are known to positively influence tumor growth, invasion, and metastasis (31). Serum levels of IL-1β, IL-6, and TNF-α have also been linked to poor CRC prognosis (32). Our *in vitro* experiments confirmed that Fn infection promoted IL-1β, IL-6, and TNF-α secretion from the myeloid cell line (THP-1 cells). These results suggest that Fn-induced colorectal tumorigenesis and poor prognosis are closely related to alterations in the immune microenvironment characterized by proinflammatory cytokine release from myeloid cells.

The colorectal tumor microenvironment comprises immunoinflammatory cells, stromal cells, secretory products, metabolites, and extracellular matrix (33). Recent studies have recognized intratumoral microbiota as integral components of this microenvironment (34). Chronic inflammation plays a critical role in cancer progression, with persistent inflammatory stimuli driving tumor malignancy (35). Specifically, the inflammatory tumor microenvironment contributes to CRC progression through mechanisms such as angiogenesis and immune regulation, involving complex interactions between tumor cells and inflammatory mediators (34). CRC progression often coincides with inflammatory responses (36). Inflammatory cells and cytokines function as active tumor-promoting factors, inducing angiogenesis and stimulating cancer cell proliferation and invasion (29, 37). Key inflammatory mechanisms in CRC include inflammasome and NF-κB pathway activation, often triggered by microbial stimuli or cytokines. Fn activates immune signaling pathways through surface molecules like FadA and Fap2, disrupting tissue homeostasis and establishing an inflammatory environment. Fn infection stimulates tumor cells to produce various cytokines, some promoting cancer cell proliferation and migration via autocrine signaling, and others affecting neighboring cells through paracrine signaling (38). Cytokines such as IL-6, IL-8, and TNF-α activate NF-κB signaling pathways to participate in CRC development (39). NF-κB activation leads to the production of proinflammatory cytokines, notably IL-6, facilitating survival and proliferation of intestinal epithelial cells, particularly in colitis-associated cancers. Additionally, NF-κB regulates TNF-α expression, frequently elevated in IBDs, colorectal adenomas, and carcinomas (40, 41). These cytokines interact with cells expressing their specific receptors, promoting tumor growth within an inflammatory microenvironment.

The inflammatory response in CRC closely relates to host immune cells, including neutrophils, macrophages, and DCs. The CRC microenvironment contains two distinct types of tumor-infiltrating immune cells: anti-tumor cells, such as natural killer cells and cytotoxic CD8^+^ T cells, and immunosuppressive cells, such as MDSCs and tumor-associated macrophages. Fn reshapes the tumor immune environment and accelerates CRC progression by increasing immunosuppressive cell populations and suppressing anti-tumor cell activity. These immune cells are intimately connected with inflammatory mediators, particularly TNF-α, IL-6, and IL-1β, which significantly influence immune responses and inflammation. Elevated expression of IL-1β, IL-6, and TNF-α has been demonstrated to promote colorectal tumor growth, invasion, and metastasis (42). IL-6, predominantly secreted by monocytes, macrophages, T and B lymphocytes, and various tumor cells, participates in CRC progression (43). IL-6 promotes T and B cell differentiation, suppresses immune responses, induces angiogenesis, and facilitates colorectal tumor cell proliferation. IL-6 activates Wnt/β-catenin signaling through IL-6/STAT3/ERK pathways, enhancing CRC cell invasion and metastasis (44). Additionally, IL-6 stimulates tumor angiogenesis by increasing vascular endothelial growth factor (VEGF) expression in CRC cells (45). IL-1β, primarily secreted by activated macrophages and lymphocytes, enhances tumor growth and metastasis through induction of inflammatory chemokines and growth factors (46, 47). Elevated IL-1β expression in the tumor microenvironment is closely associated with tumor invasion, metastasis, and angiogenesis (47). TNF-α, secreted by monocytes and macrophages, is closely linked to intestinal inflammation and colitis-associated CRC progression. TNF-α activation of NF-κB signaling creates a feedforward loop promoting CRC cell survival and proliferation. TNF-α also stimulates production of cytokines such as IL-1α and IL-6 (48). Although TNF-α can exhibit cytotoxicity towards tumor cells, it also promotes proliferation and differentiation, thereby significantly contributing to tumor development.

In this study, immunohistochemical analysis was performed to evaluate inflammatory factor expression in CRC and adjacent normal tissues. The results showed that TNF-α, IL-6, and IL-1β were weakly expressed in the cytoplasm of adjacent normal colon cells, whereas their expression levels were significantly elevated in CRC tissues. These findings suggest that inflammatory factors play important roles in CRC initiation and progression. As critical components of the inflammatory microenvironment, inflammatory factors are closely associated with immune cells. Immune cells secrete inflammatory cytokines, while inflammatory factors also regulate immune cell function and the immune microenvironment. Therefore, investigating immune cell infiltration in CRC tissues is necessary.

In this study, commonly used immune markers, including CD68, CD11C, MHCI, and MHCII, were selected for analysis. CD68 is expressed in multiple mature macrophage populations, including macrophages in the peritoneum, intestinal lamina propria, and bone marrow stroma. CD68 expression changes significantly during macrophage maturation and activation. CD11C is primarily expressed in myeloid lineage cells, including myelocytes, promyelocytes, late myelocytes, and neutrophils, and is highly expressed in tissue macrophages and monocytes. It can also be detected in NK cells, activated T cells, and lymphocyte lineages. MHCII molecules are mainly expressed on antigen-presenting cells, including B cells, monocytes/macrophages, and DCs, where they coordinate immune cell interactions during immune responses. Multiplex immunofluorescence analysis using CD45/CD11b, CD68, CD11C, MHCI, and MHCII demonstrated significantly elevated expression of these immune markers in Fn-enriched CRC tissues compared with adjacent normal tissues. These findings indicate marked enrichment of myeloid cells in Fn-enriched CRC tissues. Fn may recruit macrophages, monocytes, and lymphocytes, which subsequently secrete large amounts of inflammatory factors, thereby establishing an immune and inflammatory microenvironment favorable for CRC growth and progression.

Although Fn recruits immune cells such as macrophages, monocytes, and lymphocytes to regulate the CRC microenvironment, whether Fn directly stimulates immune cells to secrete inflammatory cytokines remained unclear. Therefore, *in vitro* experiments were performed using the myeloid cell line THP-1. THP-1 cells are widely used in studies of monocyte/macrophage-associated mechanisms and signaling pathways and can be differentiated into macrophage-like cells, making them suitable models for inflammation and immunity research. To clarify the role of Fn, myeloid cells, and inflammatory cytokines in inflammation-associated tumor progression, Fn was cocultured with THP-1 cells. After incubation, supernatants from the coculture system were collected for ELISA analysis, while uninfected THP-1 cells served as controls. The results demonstrated that TNF-α, IL-6, and IL-1β levels in the Fn-infected group were significantly higher than those in the control group. Furthermore, cytokine levels gradually increased with prolonged infection time. These findings suggest that Fn can directly stimulate THP-1 cells to secrete inflammatory cytokines, which may contribute to the inflammatory microenvironment associated with CRC. However, these *in vitro* findings do not establish that the *in vivo* effects of Fn are mediated exclusively through direct activation of myeloid cells. Previous studies have also reported the presence of IL-1β, IL-6, and TNF-α in single-cell suspension cultures derived from CRC tissues, and elevated serum levels of these cytokines have been associated with poor CRC prognosis. In addition, our 16S rRNA sequencing results showed that Fn administration was associated with marked alterations in gut microbial composition, suggesting that indirect microbiota-dependent mechanisms may also contribute to the inflammatory and tumor-promoting phenotypes observed *in vivo*. These potential mechanisms may include alterations in microbial metabolites, disruption of epithelial tight junctions and barrier integrity, increased intestinal permeability, and enhanced exposure of immune cells to microbial products. Because microbial metabolites and intestinal barrier function were not directly assessed in the present study, their contributions remain to be experimentally determined. Collectively, these findings indicate that CRC inflammatory microenvironment remodeling, characterized by the release of IL-1β, IL-6, and TNF-α from myeloid cells, may contribute to Fn-associated tumor progression together with indirect microbiota- or barrier-mediated inflammatory responses. These inflammatory factors may therefore serve as potential therapeutic targets for early intervention, providing novel strategies for CRC treatment and for overcoming immunosuppression and chemotherapy resistance.

NLRP3 has been extensively studied for its contribution to colitis and has been associated with colitis-associated CRC development (49). The tumor microenvironment is a critical determinant of tumor evolution, and inflammatory signaling pathways within this environment drive CRC progression. Inflammatory responses involved in CRC development include DNA damage induced by neutrophils, macrophages, and DCs, as well as cyclooxygenase-2 production. In addition, pathogen invasion of the intestinal mucosa activates immune cells and induces secretion of cytokines and growth factors that sustain inflammatory responses. Persistent inflammation promotes epithelial proliferation, angiogenesis, and apoptosis inhibition, ultimately contributing to carcinogenesis (50). Macrophages and T cells secrete proinflammatory cytokines such as IL-6 and TNF-α, promoting differentiation of proinflammatory Th17 cells. Persistent Th17 activation and elevated IL-17 and IL-22 levels have been associated with poor survival outcomes. Increased serum IL-6 and TNF-α levels have also been used as prognostic indicators of poor survival in CRC patients (51). Cytokines are small proteins involved in cell communication and can function through autocrine, paracrine, or endocrine mechanisms. They are essential for maintaining homeostasis and host defense, and many cytokines participate in inflammatory responses. IL-1β, one of the most extensively studied cytokines, is secreted by multiple cell types during inflammation, infection, tissue injury, and cancer (52). Cytokine and chemokine secretion can also be induced by conformational changes in inflammasome complexes, leading to caspase-1 activation (53). As a member of the NLRP inflammatory protein family, NLRP3 is primarily expressed in monocytes, macrophages, epithelial cells, and adaptive immune cells such as T cells (36). Immunohistochemical analysis in the present study further confirmed that NLRP3 and IL-1β expression levels were significantly elevated in cancer tissues with high Fn abundance compared with adjacent normal tissues.

MDSCs play a critical role in tumor progression. Various immune cells, cytokines, and mediators contribute significantly to tumorigenesis, including tumor initiation, progression, and metastasis. CD45 is a leukocyte common antigen typically present on white blood cells, whereas CD11b is primarily expressed on granulocytes, monocytes, and macrophages (54). Studies have demonstrated that the NLRP3 inflammasome is localized in bone marrow-derived macrophages (BMDMs), and its expression can be detected within CD45^+^ immune cells in tumor lysates (55). Using multiplex immunofluorescence, we evaluated CD45^+^ and CD11b^+^ immune cell expression in each tissue group. Results indicated significantly higher expression of CD45^+^ and CD11b^+^ cells in Fn-enriched cancer tissues compared to adjacent normal tissues, where expression was nearly absent. These observations suggest that Fn may contribute to CRC progression by selectively amplifying myeloid immune cells and reshaping the tumor microenvironment.

Fn infection can upregulate inflammatory factors (e.g., IL-1β, IFN-γ, TNF-α), thereby altering the tumor microenvironment. IL-1β activation involves two processes: transcription of pro-IL-1β regulated by NF-κB, and maturation regulated by inflammasomes (56). To further investigate the link between NLRP3 and Fn-mediated CRC, we co-cultured Fn with BMDMs for 4 and 8 hours and collected culture supernatants to detect NLRP3 inflammasome and IL-1β expression via immunoblotting. The results demonstrated weak expression of NLRP3 and IL-1β in supernatants without Fn (0 h co-culture). However, Fn presence (4 h and 8 h co-culture) significantly increased NLRP3 inflammasome and IL-1β expression in a time-dependent manner. These findings suggest Fn infection recruits immune cells and promotes inflammatory factor secretion, aiding tumor cells in reconstructing the immune microenvironment favorable for CRC proliferation.

In this study, the effects of Fn were compared with a vehicle control (saline) and another oral bacterium, *Streptococcus mutans* (S.M), on tumor growth (**Figure 2**). Although *S. mutans* administration also increased tumorigenesis compared with the control, its effect was markedly weaker than that induced by Fn (**Figures 2I, J**). Moreover, 16S rRNA sequencing analysis revealed distinct alterations in gut microbiota composition induced by Fn, characterized by increased pro-inflammatory taxa such as Desulfobacterota and reduced beneficial Akkermansiaceae. Several of these alterations were more pronounced in the Fn-treated group than in the *S. mutans*-treated group (**Figures 3C–F**, **4C–F**). These findings demonstrate that Fn produced a stronger pro-tumorigenic and inflammatory phenotype than *S. mutans* under the conditions examined. However, this comparison does not establish that NLRP3 activation or the associated tumor-promoting mechanism is unique to *Fn*. Importantly, *S. mutans* is Gram-positive, whereas Fn is Gram-negative and contains LPS. **L**PS may activate TLR4–NF-κB signaling and provide a priming signal that increases the expression of NLRP3 and pro-IL-1β. Therefore, other Gram-negative intestinal or oral pathobionts may potentially activate similar inflammatory pathways. The present study should consequently be interpreted as demonstrating an Fn-associated, rather than strictly Fn-specific, mechanism. Comparisons with additional Gram-negative bacteria, together with experiments using purified LPS or TLR4 inhibition, will be required to determine the degree of pathogen specificity. Nevertheless, our results demonstrate that Fn activates the NLRP3/caspase-1/IL-1β pathway in myeloid cells and that NLRP3 deficiency markedly attenuates Fn-associated tumor growth.

Although our study establishes Fn-mediated activation of the NLRP3 inflammasome as indispensable for promoting CRC via the Fn/NLRP3/IL-1β axis, the precise upstream mechanisms by which Fn initiates NLRP3 upregulation merit further exploration. Based on current findings and existing literature, several potential pathways are plausible. Firstly, NF-κB activation serves as a canonical priming signal for NLRP3 and pro-IL-1β transcription. Fn surface virulence factors, including FadA and LPS, activate pattern-recognition receptors such as TLR4, subsequently triggering NF-κB signaling (23, 39). Therefore, Fn surface molecules might provide initial transcriptional priming of NLRP3. Additionally, Fn-secreted outer membrane vesicles (OMVs) or metabolites may represent another critical mechanism. OMVs containing PAMPs, such as LPS and lipoproteins, could directly activate host cells, providing efficient signals for inflammasome assembly. Moreover, Fn-induced dysbiosis significantly alters gut microbial composition, increasing pro-inflammatory taxa such as Desulfobacterota. Such dysbiosis may generate specific microbial metabolites (e.g., secondary bile acids, hydrogen sulfide) that activate NLRP3 or serve as DAMPs. Future studies should directly test whether Fn-derived OMVs or culture filtrates activate NLRP3 in myeloid cells. Additionally, metabolomic profiling of Fn or gut contents from Fn-treated mice could identify specific metabolites responsible for observed inflammatory reprogramming. Elucidating these mechanisms, via surface factor interactions, vesicle-mediated delivery, or metabolite-induced signaling, will enhance understanding of the Fn/NLRP3/IL-1β axis and potentially uncover novel therapeutic targets for preventing Fn-driven carcinogenesis.

The integrated Fn-NLRP3-myeloid cell-IL-1β signaling axis established herein distinguishes this study from previous work that reported these components individually. This axis offers actionable targets for clinical translation. Fn abundance in tissues or feces could serve as a noninvasive biomarker for early CRC screening and prognosis stratification. Pharmacological inhibition of NLRP3 or neutralization of IL-1β may represent promising adjunctive strategies for blocking Fn-driven tumor progression, especially in patients with high Fn colonization. Our findings also support microbiota-targeted interventions (e.g., Fn eradication) as potential approaches for CRC prevention and treatment.

In summary, our study demonstrates that Fn enrichment in CRC tissues promotes tumor progression through recruitment of bone marrow-derived myeloid cells, activation of the NLRP3 inflammasome, and establishment of a pro-inflammatory microenvironment conducive to colorectal tumor growth. This Fn-NLRP3 signaling axis deepens the understanding of microbiota-mediated carcinogenesis and provides novel biomarkers and therapeutic targets for clinical management of CRC. Future research should investigate Fn interactions with other gut microbes and host components to optimize translational therapeutic strategies.

## Conclusion

Fn drives CRC progression through an NLRP3-dependent mechanism by selectively expanding bone marrow-derived myeloid immune cells and reshaping the pro-tumor inflammatory microenvironment. This integrated signaling axis represents a unique advance compared to prior studies that investigated these components separately.

## Data Availability

The datasets generated for this study can be found in the NCBI Repository; Accession number: PRJNA1507281.
